# miR-338-3p Is Regulated by Estrogens through GPER in Breast Cancer Cells and Cancer-Associated Fibroblasts (CAFs)

**DOI:** 10.3390/cells7110203

**Published:** 2018-11-09

**Authors:** Adele Vivacqua, Anna Sebastiani, Anna Maria Miglietta, Damiano Cosimo Rigiracciolo, Francesca Cirillo, Giulia Raffaella Galli, Marianna Talia, Maria Francesca Santolla, Rosamaria Lappano, Francesca Giordano, Maria Luisa Panno, Marcello Maggiolini

**Affiliations:** 1 Department of Pharmacy, Health and Nutritional Sciences, University of Calabria, 87036 Rende, Italy; annasebastiani86@gmail.com (A.S.); damianorigiracciolo@yahoo.it (D.C.R.); francesca89cirillo@libero.it (F.C.); giulia.r.galli@gmail.com (G.R.G.); mariannatalia11@gmail.com (M.T.); m.f.s@hotmail.it (M.F.S.); lappanorosamaria@yahoo.it (R.L.); francesca.giordano@unical.it (F.G.); mamissina@yahoo.it (M.L.P.); 2 Regional HospitalCosenza, 87100 Cosenza, Italy; annamariamiglietta@virgilio.it

**Keywords:** breast cancer, CAFs, estrogens, GPER, miR-338-3p, c-Fos, Cyclin D1

## Abstract

Estrogens acting through the classic estrogen receptors (ERs) and the G protein estrogen receptor (GPER) regulate the expression of diverse miRNAs, small sequences of non-coding RNA involved in several pathophysiological conditions, including breast cancer. In order to provide novel insights on miRNAs regulation by estrogens in breast tumor, we evaluated the expression of 754 miRNAs by TaqMan Array in ER-negative and GPER-positive SkBr3 breast cancer cells and cancer-associated fibroblasts (CAFs) upon 17β-estradiol (E2) treatment. Various miRNAs were regulated by E2 in a peculiar manner in SkBr3 cancer cells and CAFs, while miR-338-3p displayed a similar regulation in both cell types. By METABRIC database analysis we ascertained that miR-338-3p positively correlates with overall survival in breast cancer patients, according to previous studies showing that miR-338-3p may suppress the growth and invasion of different cancer cells. Well-fitting with these data, a miR-338-3p mimic sequence decreased and a miR-338-3p inhibitor sequence rescued the expression of genes and the proliferative effects induced by E2 through GPER in SkBr3 cancer cells and CAFs. Altogether, our results provide novel evidence on the molecular mechanisms by which E2 may regulate miR-338-3p toward breast cancer progression.

## 1. Introduction

Estrogens play a crucial role in diverse pathophysiological conditions, including cancer [1]. The action of estrogens are mainly mediated by the classic estrogen receptors (ERs) [2], however several data have also indicated that the G protein estrogen receptor (GPER) may trigger a network of transduction pathways toward the progression of several types of tumors [3,4,5,6,7,8]. Among numerous biological targets, estrogens may modulate the expression of diverse microRNAs (miRNAs) [6], which are small non-coding RNA molecules of 22–25 nucleotides [9]. In particular, miRNAs inhibit the expression of certain genes at both transcriptional and post-transcriptional levels binding to complementary sequences located within the 3’ untranslated region (UTR) of target mRNAs [10,11]. Therefore, miRNAs may be involved in important biological processes, including cancer development [12,13,14,15,16,17,18,19,20]. The involvement of ERs in miRNA regulation by estrogens has been established [6]. Likewise, it has been also reported that GPER may regulate the expression of certain miRNAs in normal and cancer cell contexts characterized by the presence or absence of ERs [21,22,23,24,25].

MiR-338-3p is a highly conserved gene located on the chromosome 17q25 and precisely on the 7^th^ intron of the apoptosis-associated tyrosine kinase (AATK) [26,27]. MiR-338-3p, initially identified as a brain specifically expressed miRNA, has been involved in the formation of basolateral polarity and regulation of axonal respiration [28,29]. Various studies have also shown that miR-338-3p is downregulated in many types of malignancies, hence suggesting its potential role in tumor progression [30,31,32,33,34]. Nevertheless, the biological function of miR-338-3p and its prognostic significance remains to be fully understood.

In this present study we provide novel insights into the ability of estrogens to regulate miR-338-3p expression and function through GPER in ER-negative breast cancer cells and cancer associated fibroblasts (CAFs), which are main components of the tumor microenvironment [35,36]. On the basis of our findings miR-338-3p may be included among the miRNAs involved in breast tumor development.

## 2. Materials and Methods

### 2.1. Reagents

17β-estradiol (E2) was purchased from Sigma-Aldrich Corp. (Milan, Italy); rel-1-[4-(6-bromo-1,3-benzodioxol-5-yl)-3aR,4S,5,9bS-tetrahydro-3H-cyclopenta[c]quinolin-8-yl]-ethanone (G-1) was obtained from Tocris Bioscience (Space, Milan, Italy). All compounds were solubilized in dimethyl sulfoxide (DMSO).

### 2.2. Cell Cultures

Breast cancer cell line SkBr3 (ER-negative and GPER-positive) was obtained from ATCC (Manassas, VA, USA), used less than six months after revival and routinely tested and authenticated according to the ATCC suggestions. CAFs (ER-negative and GPER-positive) were extracted from invasive mammary ductal carcinomas obtained from mastectomies. Briefly, samples were cut into smaller pieces (1–2 mm diameter), placed in digestion solution (400 IU collagenase I, 100 IU hyaluronidase, and 10% FBS, containing antibiotic and antimycotic solution) and incubated overnight at 37 °C. The cells were then separated by differential centrifugation at 90× *g* for 2 min. Supernatant containing fibroblasts was centrifuged at 485× *g* for 8 min; the pellet obtained was suspended in fibroblasts growth medium (Medium 199 and Ham’s F12 mixed 1:1 and supplemented with 10% FBS) and cultured at 37 °C in 5% CO_2_. Primary cells cultures of breast fibroblasts were characterized by immunofluorescence. Briefly cells were incubated with human anti-vimentin (V9, sc-6260) and human anti-cytokeratin 14 (LL001 sc-53253), both from Santa Cruz Biotechnology (DBA, Milan, Italy) (data not shown). To characterize fibroblasts activation, we used anti-fibroblast activated protein α (FAPα) antibody (SS-13, sc-100528; Santa Cruz Biotechnology, DBA, Milan, Italy) (data not shown). Signed informed consent from all the patients was obtained and samples were collected, identified and used in accordance with approval by the Institutional Ethical Committee Board (Regional Hospital, Cosenza, Italy). Cell types were grown in a 37 °C incubator with 5% CO_2_. SkBr3 breast cancer cells were maintained in RPMI-1640 without phenol red supplemented with 10% fetal bovine serum (FBS) and 100 μg/mL of penicillin/streptomycin (Gibco, Life Technologies, Milan, Italy). CAFs were cultured in a mixture of MEDIUM 199 and HAM’S F-12 (1:1) supplemented with 10% FBS and 100 μg/mL of penicillin/streptomycin (Gibco, Life Technologies, Milan, Italy). Cells were switched to medium without serum the day before experimental analysis.

### 2.3. RNA Extraction

Cells were maintained in regular growth medium and then switched to medium lacking serum before performing the indicated assays. Total RNA was extracted from cultured cells using miRVana Isolation Kit (Ambion, Life Technologies, Milan, Italy) according to the manufacturer’s recommendations. The RNA concentrations were determined using Gene5 2.01 Software in Synergy H1 Hybrid Multi-Mode Microplate Reader (BioTek, AHSI, Milan, Italy).

### 2.4. miRNA Expression Profiling

TaqMan™ Array Human MicroRNA A+B Cards Set v3.0 was used for global miRNA profiling. The panel includes two 384-well microfluidic cards (human miRNA pool A and pool B) that contain primers and probes for 754 different miRNAs in addition to small nucleolar RNAs that function as endogenous controls for data normalization. Equal quantity (100 ng) of RNA extracted from SkBr3 breast cancer cells and CAFs treated with vehicle or 100 nM E2 for 4 h was reverse-transcribed for cDNA synthesis using the Megaplex RT Primer Pool A or B and the TaqMan MicroRNA Reverse Transcription kit (Applied Biosystems).in a final volume of 7.5 μL (Applied Biosystems, Milan, Italy). The reverse transcription reaction was incubated for 2 min at 16 °C, 1 min at 42 °C and 1 s at 50 °C for 40 cycles, followed by 5 min at 85 °C to deactivate the enzyme. The cDNA obtained was pre-amplified using Megaplex Preamp primer pool A or B and TaqMan PreAmp Master Mix 2X in a final volume of 25 μL using the same temperature conditions above described. The product was diluted 1:4 in TE 0.1X, to which were added TaqMan Universal Master Mix no UNG 2X and nuclease free water. 100 μL of the sample/master mix for each multiplex pool were loaded into fill reservoirs on the microfluidic card. The array was then centrifuged, mechanically sealed with the Applied Biosystems sealer device and run on QuantStudio 6&7 Flex Real Time PCR System (Applied Biosystems, Life Technologies, Milan, Italy). The raw array data were analysed by DataAssist^TM^. The baseline was set automatically, while the threshold was set manually at 0.2. Samples that had Ct values>32 were removed from the analysis. Each miRNA was normalized against the mean of the four RNU6B and its expression was then assessed in the E2 treated cells against the vehicle treated cells using the 2^−ΔΔCT^ method [37]. miRNAs showing an increased value of 2-fold expression and a 50% reduction respect to vehicle-treated cells were selected. Venn diagram was obtained by http://bioinformatics.psb.ugent.be/cgibin/liste/Venn/calculate_venn.htpl.

### 2.5. Analysis of Public Data Set from METABRIC and Kaplan-Meier Plotter

Prognostic values of miR-338-3p levels, using METABRIC data set, were analyzed by Kaplan–Meier survival curves of breast cancer patients, using Kaplan-Meier Plotter (www.kmplot.com/analysis) [38]. Log-rank test was used for statistical analysis.

### 2.6. Real Time-PCR

cDNA for miRNA expression was synthesized from 100 ng of total RNA using the TaqMan microRNA Reverse Transcription Kit (Applied Biosystems, Life Technologies, Milan, Italy). The expression levels of miR-338-3p were quantified by TaqMan microRNA Assay Kit (Applied Biosystems, Milan, Italy), using the primers for the internal control RNU6B (assay ID 001093) and miR-338-3p (assay ID 002252). In order to measure the mRNA levels of c-Fos and Cyclin D1, 3µg of total RNA were reversely transcribed using the murine leukemia virus reverse transcriptase (Life Technologies, Milan, Italy), as indicated by the manufacturer. The quantitative PCR was performed using SYBR Green PCR Master Mix (Applied Biosystems, Life Technologies, Milan, Italy). Specific primers for Actin, which was used as internal control, c-Fos and Cyclin D1 genes were designed using Primer Express version 2.0 software (Applied Biosystems Inc, Milano, Italy). The sequences were as follows: Actin Fwd: 5′-AAGCCAACCCCACTTCTCTCTAA-3′ and Rev: 5′-CACCTCCCCTGTGTGGACTT-3′; c-Fos Fwd: 5′-CGAGCCCTTTGATGACTTCCT-3′ and Rev: 5′-GGAGCGGGCTGTCTCAGA-3′; Cyclin D1 Fwd: 5′-CCGTCCATGCGGAAGATC-3′ and Rev: 5′-ATGGCCAGCGGGAAGAC-3′. All experiments were performed in triplicate using QuantStudio 6&7 Flex Real Time PCR System (Applied Biosystems, Life Technologies, Milan, Italy). Data were normalized to the geometric mean of housekeeping gene to control the variability into expression levels and fold changes were calculated by relative quantification compared to respective scrambled controls [32].

### 2.7. Bioinformatic Tools

The sites miRNAbase (http://www.miRNAbase.org), Targetscan (http://www.targetscan.org) and miRDip (http://ophid.utoronto.ca/mirDIP/) were used to identified miR-338-3p target genes.

### 2.8. Constructs and Transfections

The negative control (miR-Ctrl), the miR-338-3p mimic (miR-338-3p m) (ID MC10716) and miR-338-3p inhibitor (miR-338-3p i) (ID MH10716) sequences were purchased from Ambion (Life Technologies, Milan, Italy) and transfected into the cells 48 h before the treatments, using X-treme GENE 9 DNA Transfection Reagent (Roche Diagnostics, Sigma-Adrich, Milan, Italy). Silencing of GPER expression was obtained by using the construct previously described [39]. The plasmid DN-Fos, which encodes a c-Fos mutant that heterodimerizes with c-Fos dimerization partners but does not allow DNA binding, was a kind gift from Dr. C. Vinson (NIH, Bethesda, MD, USA).

### 2.9. Western Blotting

Cells were maintained in complete medium before the transfection assays, which are performed in medium without serum for 48 h and then treated as indicated. Cells were lysed in RIPA buffer containing a mixture of protease inhibitors. Equal amounts of protein extract were resolved on SDS-polyacrylamide gel, transferred to a nitrocellulose membrane (Amersham Biosciences, Italy), probed overnight at 4 °C with antibodies against: c-Fos (E-8, sc-166940) and β-Actin (AC-15, sc-69879) (Santa Cruz Biotechnology, DBA, Italy), GPER (AB137479) (Abcam, Euroclone, Milan, Italy) and Cyclin D1 (Origene, DBA, Milan, Italy). Proteins were detected by horseradish peroxidase-linked secondary antibodies (Biorad, Milan, Italy) and revealed using the chemiluminescent substrate for western blotting Westar Nova 2.0 (Cyanagen, Biogenerica, Catania, Italy).

### 2.10. Luciferase Assays

Cells were seeded in regular growth medium into 24-well plates. The next day the growth medium was replaced with medium lacking serum and the transfection was performed using X-tremeGene9 reagent, as recommended by the manufacturer (Roche Diagnostics), with a mixture containing Cyclin-D1-luc, the internal control pRL-TK and miR-Ctrl, miR-338-3p m, alone or in presence of miR-338-3p i, shGPER, DN-Fos as indicated. The cells were treated overnight with 100 nM of E2 or G1. Luciferase activity was measured using the Dual Luciferase kit (Promega, Milan, Italy) according to the manufacturer’s instructions. Firefly luciferase values were normalized to the internal transfection control provided by the Renilla luciferase activity. The normalized relative light unit (RLU) values obtained from cells transfected with respective scrambled controls were set as 1-fold induction upon which the activity induced by the treatment was calculated.

### 2.11. Cell Proliferation Assays

For quantitative proliferation assay, cells (1 × 10^4^) were seeded in 24-well plates in regular growth medium. Cells were washed, once they had attached, and then incubated in medium containing 2.5% charcoal stripped fetal bovine serum, before the transfection with 25 nM miR-338-p m and 50 nM miR-338-3p i, as indicated. Transfection was renewed every 2 day, while the cells were treated every day. Evaluation of cell growth was performed on day 6 using automatic counter (Countess™-Invitrogen).

### 2.12. Cell Cycle Analysis

To analyze cell cycle distribution, CAFs were cultured in regular medium and shifted in medium containing 2.5% charcoal-stripped FBS at the 70% confluence. Next, miRNA sequences as indicated were added to cells using X-treamGene9 reagent (Roche Diagnostics, Milan, Italy). After 24 h, 100 nM E2 or 100 nM G-1 were put in the medium for additional 24 h. Cells were pelleted, once washed with phosphate buffered saline and stained with a solution containing 50 µg/mL propidium iodide in 1 x PBS (PI), 20 U/mL RNAse-A and 0.1% Triton (Sigma-Aldrich, Milan, Italy). The DNA content was measured using a FACScan flow cytometer (Becton Dickinson, Mountain View, CA, USA) and the data acquired using CellQuest software. Cell cycle profiles were determined using ModFit LT. The proportion of the cells in G0/G1, S and G2/M phases was each estimated as a percentage of the total events (10,000 cells).

### 2.13. Statistical Analysis

Data were analyzed by one-way ANOVA with Dunnett’s multiple comparisons where applicable, using GraphPad Prism version 6.01 (GraphPad Software, Inc., San Diego, CA, USA). *p* < 0.05 (*) was considered statistically significant.

## 3. Results

### 3.1. miRNAs Expression by E2 in SkBr3 Cancer Cells and CAFs

**Figure 1:**
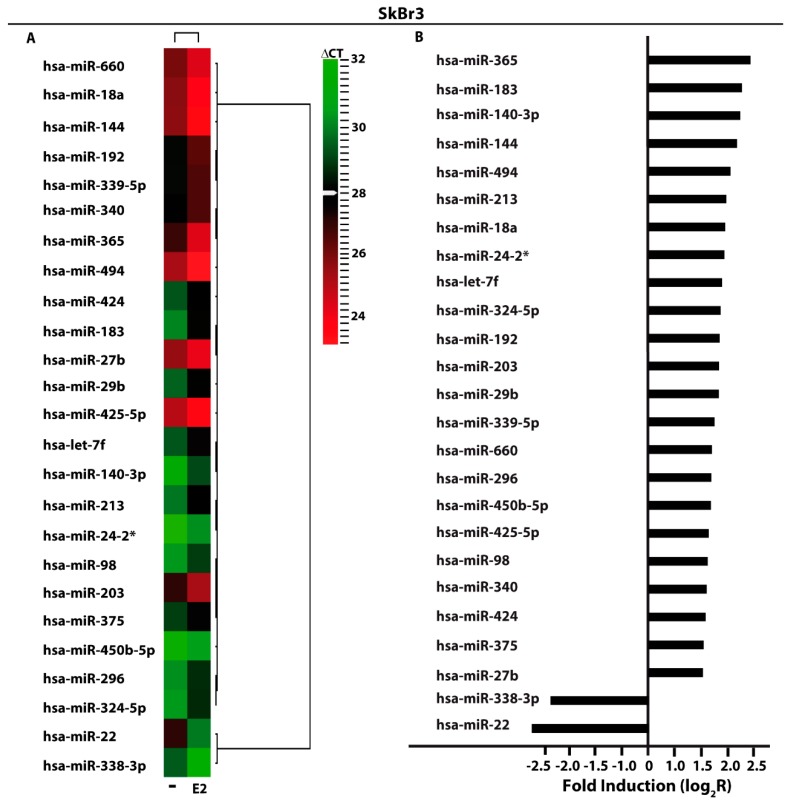
E2-modulated miRNAs expression in SkBr3 breast cancer cells. (**A**) Heat Map representation of E2-modulated miRNAs in SkBr3 cancer cells treated with 100 nM E2 for 4 h and analyzed by TaqMan Low-Density Array Human miRNA. Row represents a miRNA and column represents the treatment used. Each column is illustrated according to a color scale from green (low expression) to red (high expression). The distance measured is Euclidean Distance and the clustering method is complete linkage. Dendrograms of clustering analysis for miRNAs and samples are displayed on the top and right, respectively. (**B**) Up- and down-regulated miRNAs in SkBr3 breast cancer cells upon E2 stimulation. The values are indicated as log2 fold change (R) calculated respect to vehicle (-).

**Figure 2:**
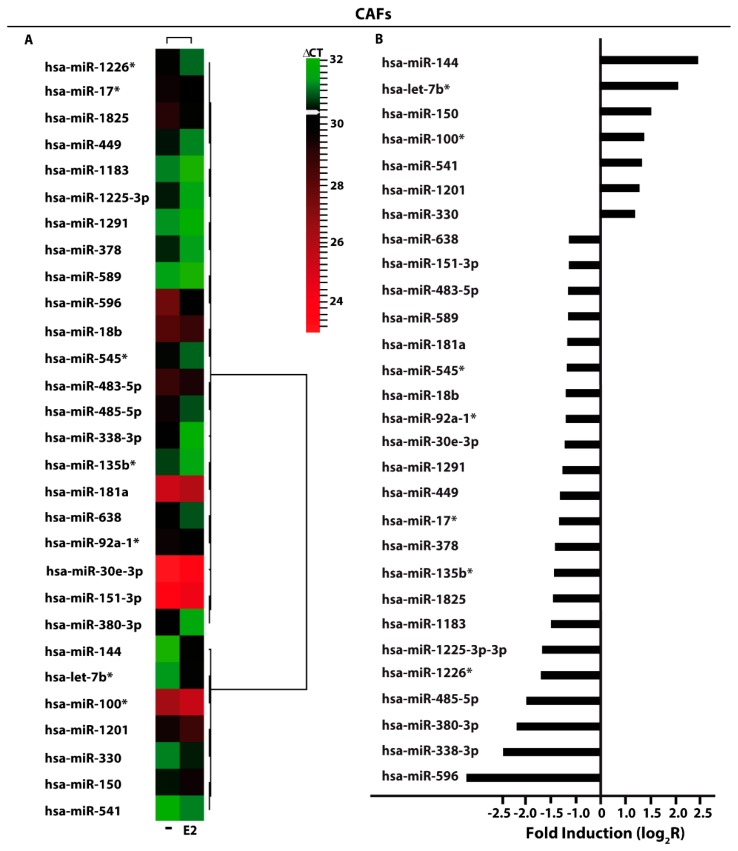
E2-modulated miRNAs expression in CAFs. (**A**) Heat Map representation of E2-modulated miRNAs in CAFs treated with 100 nM E2 for 4 h and analyzed by TaqMan Low-Density Array Human miRNA. Row represents a miRNA and column represents the treatment used. Each column is illustrated according to a color scale from green (low expression) to red (high expression). The distance measured is Euclidean Distance and the clustering method is complete linkage. Dendrograms of clustering analysis for miRNAs and samples are displayed on the top and right, respectively. (**B**) Up- and down-regulated miRNAs in CAFs upon E2 stimulation. The values are indicated as log2 fold change (R) calculated respect to vehicle (-).

**Figure 3:**
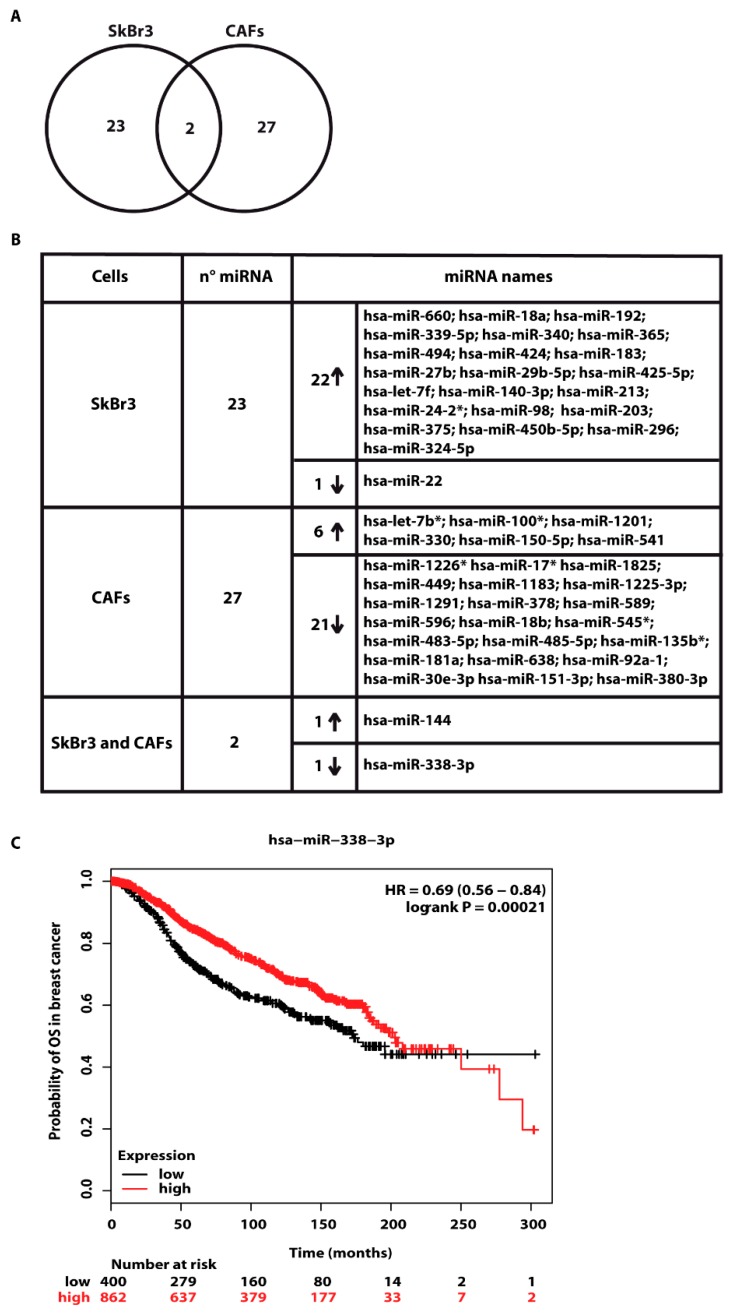
Exclusive and shared expression of miRNAs between SkBr3 and CAFs. (**A**) Venn Diagram of E2-modulated miRNAs in SkBr3 cancer cells and CAFs. (**B**) Up and down-regulated miRNAs by 100 nM E2 treatment for 4 h in SkBr3 cancer cells and CAFs. (**C**) The expression of miR-338-3p is associated with higher overall survival in breast cancer patients. The evaluation was performed by Kaplan–Meier Plotter (http://www.kmplot.com). Statistical analysis was made using the log-rank test.

In order to provide novel insights on the action of estrogens toward miRNAs modulation in breast cancer, the ER-negative SkBr3 breast cancer cells and CAFs were treated with 100 nM E2 for 4 h and then analyzed by TaqMan™ Array Human MicroRNA. A total amount of 754 miRNAs involved in diverse pathophysiological conditions (www.thermofisher.com/order/catalog/product/4444913) were evaluated, thereafter we focused our attention on miRNAs displaying a Ct< 32 along with at least 2 fold increase or 50% reduction upon E2 exposure respect to vehicle-treated cells. On the basis of these criteria, we identified 25 and 29 E2-regulated miRNAs in SkBr3 cancer cells (Figure 1A) and CAFs (Figure 2A), respectively. In particular, in SkBr3 cancer cells 23 miRNAs were up-regulated and 2 miRNAs were down-regulated by E2 treatment (Figure 1B). As it concerns CAFs, among the 29 E2-regulated miRNAs, 7 showed an increase and 22 a reduction upon E2 stimulation (Figure 2B). To identify unique and shared E2-regulated miRNAs in both cell types, we then calculated a Venn diagram. SkBr3s cancer cells and CAFs shared only the expression of 2 miRNAs (Figure 3A), namely miR-144 and miR-338-3p, which exhibited a similar response (Figure 3B). Considering that in our previous studies we evaluated the role of miR-144 in tumor cell growth [25], in the present investigation we aimed to determine the mechanisms leading to the estrogen regulation of miR-338-3p and its action in breast cancer. Hence, we began our study ascertaining that miR-338-3p expression correlates positively with the overall survival in 1283 breast tumor patients, as reported in the Molecular Taxonomy of Breast Cancer International Consortium (METABRIC) database [40] (Figure 3C). Nicely fitting with these findings, previous evidence has suggested that miR-338-3p may function as a tumor suppressor in certain malignancies including breast cancer [30,31,32,33,34].

### 3.2. GPER Is Involved in the Regulation of miR-338-3p by E2 and G-1 in SkBr3 Cancer Cells and CAFs

**Figure 4:**
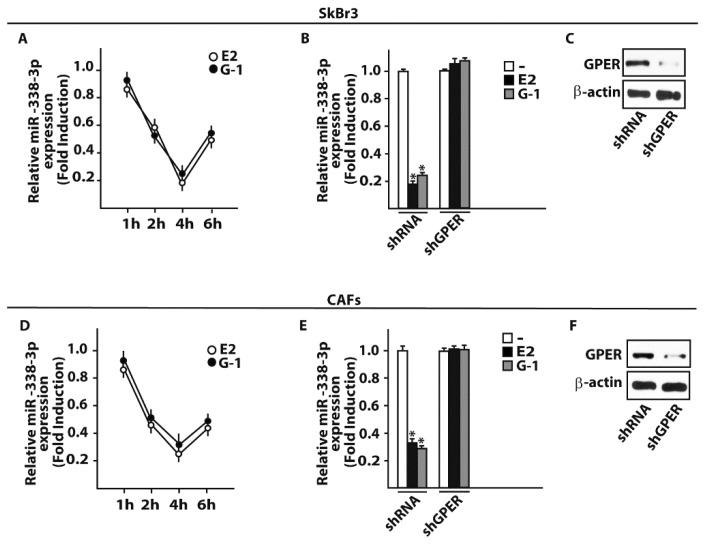
E2 and G-1 down-regulate miR-338-3p levels in SkBr3 cancer cells and CAFs. SkBr3 breast cancer cells (**A**) and CAFs (**D**) were stimulated with 100 nM E2 or 100 nM G-1 as indicated and analyzed by RT-PCR. Each point is plotted as fold changes of cells receiving treatments respect to cells treated with vehicle (-) and represents the mean ± SD of three independent experiments performed in triplicate. MiR-338-3p expression upon a 4 h treatment with 100 nM E2 or 100 nM G-1 in SkBr3 cells (**B**) and CAFs (**E**) previously transfected with shRNA or shGPER for 48 h. Each column represents the mean ± SD of three independent experiments performed in triplicate. Efficacy of GPER silencing in SkBr3 cells (**C**) and CAFs (**F**). β-actin serves as a loading control. (*) indicates *p* < 0.05, for cells receiving treatments vs cells treated with vehicle.

**Figure 5:**
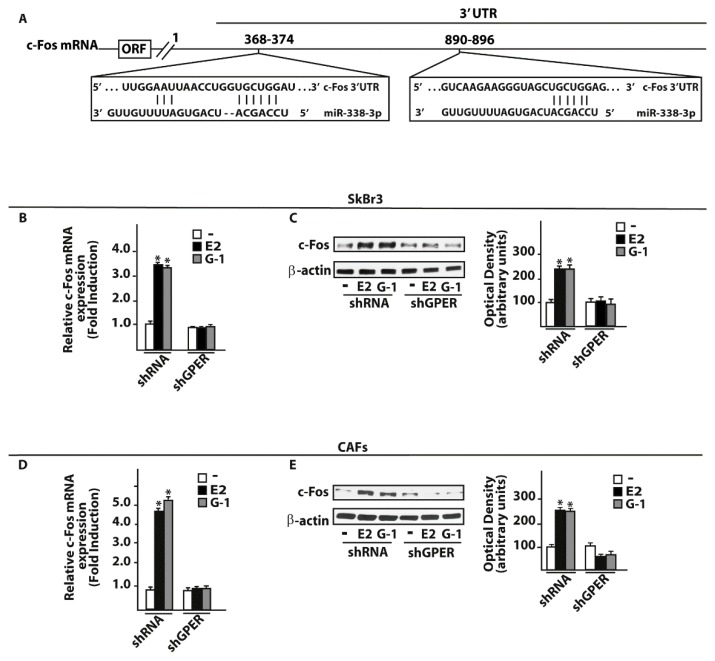
c-Fos is a target gene of miR-338-3p. (**A**) Schematic alignment between the miR-338-3p sequence and the 3’-UTR mRNA region of c-Fos. mRNA expression of c-Fos in SkBr3 cancer cells (**B**) and CAFs (**D**) transfected with shRNA or shGPER for 48 h and then treated for 4 h with 100 nM E2 or 100 nM G-1. Each column represents the mean ± SD of three independent experiments performed in triplicate. c-Fos protein expression in SkBr3 cancer cells (**C**) and CAFs (**E**) transfected with shRNA or shGPER for 48 h and then treated for 4 h with 100 nM E2 or 100 nM G-1. Side panels show densitometry analysis of the blots normalized to the loading control β-actin.

**Figure 6:**
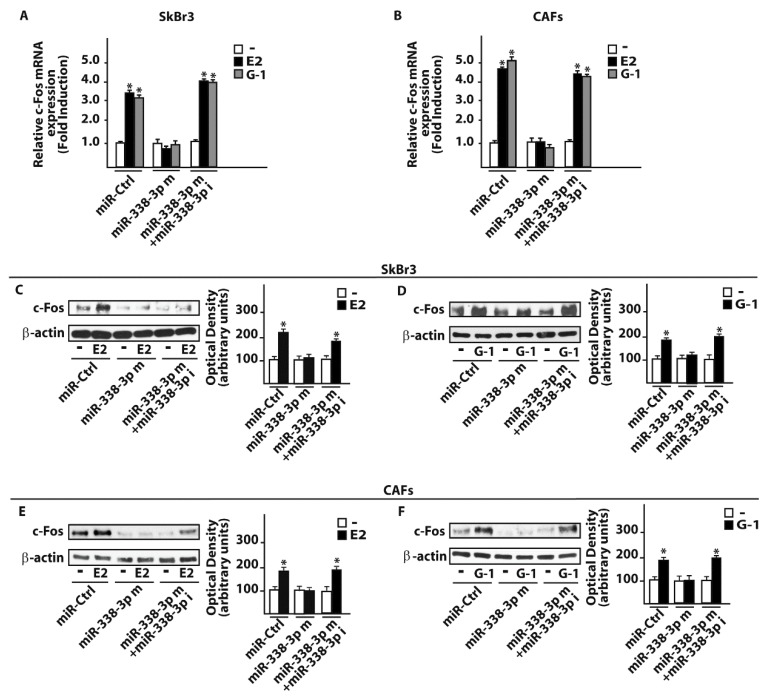
miR-338-3p prevents c-fos induction by E2 and G-1 in SkBr3 cancer cells and CAFs. mRNA levels of c-Fos in SkBr3 cancer cells (**A**) and CAFs (**B**) transfected for 48 h with 25 nM miR-Ctrl or miR-338-3p mimic (miR-338-3p m) in combination or not with 50 nM miR-338-3p inhibitor (miR-338-3p i) and then treated for 4 h with 100 nM E2 or 100 nM G-1. Each column represents the mean ± SD of three independent experiments performed in triplicate. c-Fos protein levels in SkBr3 cancer cells (**C, D**) and CAFs (**E, F**) transfected for 48 h with 25 nM miR-Ctrl or miR-338-3p mimic (miR-338-3p m) in combination or not with 50 nM miR-338-3p inhibitor (miR-338-3p i) and then stimulated for 4 h with 100 nM E2 or 100 nM G-1. Side panels show densitometry analysis of the blots normalized to the loading control β-actin. (*) indicates *p* < 0.05, for cells receiving treatments vs cells treated with vehicle (-).

On the basis of the aforementioned results, we then attempted to define the molecular mechanisms involved in the estrogenic regulation of miR-338-3p performing a time-course study upon 100 nM of E2 and 100 nM of the selective GPER ligand G-1. Worthy, the inhibitory effects of E2 and G-1 on miR-338-3p expression were no longer evident silencing GPER in SkBr3 cancer cells (Figure 4A–C) and in CAFs (Figure 4D–F). Thereafter, we aimed to identify putative target genes of miR-338-3p by a bioinformatic analysis of available algorithms (http://ophid.utoronto.ca/mirDIP; http://www.microrna.org; http://www.targetscan.org). Among others, two putative target sequences of miR-338-3p located within the 3’-UTR of the oncogene c-Fos were found (Figure 5A). According to our previous studies showing that estrogens regulate c-Fos levels in diverse cancer cell types [41,42,43,44], the induction of c-Fos mRNA and protein expression upon a 4 h treatment with 100 nM E2 and 100 nM G-1 was abolished silencing GPER in SkBr3 cancer cells (Figure 5B,C) and CAFs (Figure 5D,E). Next, we found that in SkBr3 cells and CAFs transfected for 48 h with a miR-338-3p mimic sequence, the treatment for 4 h with 100 nM E2 and 100 nM G-1 is no longer able to induce c-Fos mRNA and protein levels, a response rescued transfecting the miR-338-3p mimic sequence in combination with a miR-338-3p inhibitor sequence (Figure 6A–F).

### 3.3. miR-338-3p Triggers Inhibitory Effects on the Proliferation Induced by E2 and G-1

**Figure 7:**
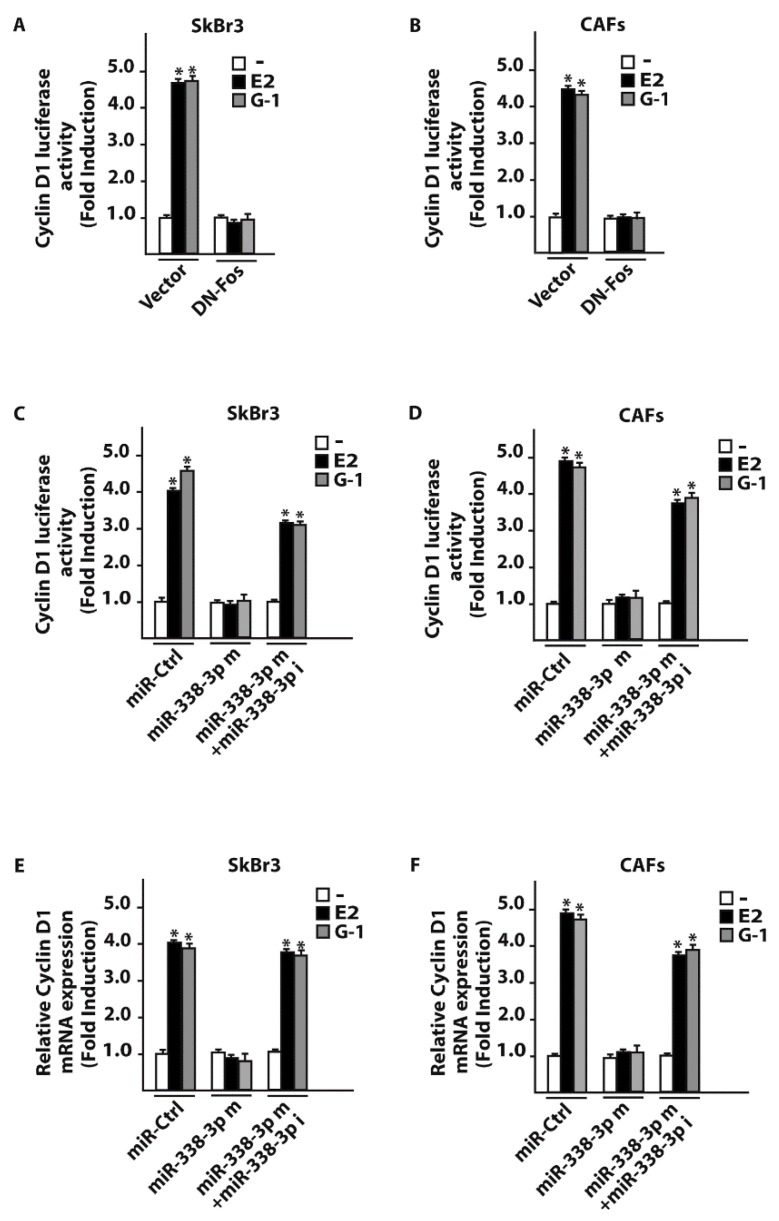
c-Fos and miR-338-3p are involved in Cyclin D1 regulation in SkBr3 cancer cells and CAFs. Luciferase activity of Cyclin D1 reporter gene in SkBr3 cancer cells (**A**) and CAFs (**B**) transfected for 8 h with a vector or a dominant-negative c-Fos construct (DN-Fos) before treatment with 100 nM of E2 and 100 nM G-1 for 18 h. Luciferase activity of Cyclin D1 reporter gene in SkBr3 cancer cells (**C**) and CAFs (**D**) transfected for 24 h with 25 nM miR-Ctrl or miR-338-3p mimic (miR-338-3p m) in combination or not with 50 nM miR-338-3p inhibitor (miR-338-3p i) before treatment for 18 h with 100 nM E2 or 100 nM G-1. The luciferase activity was normalized to the internal transfection control, values of cells receiving vehicle (-) were set as 1-fold induction upon which the activity obtained upon the indicated treatments was calculated. mRNA expression of Cyclin D1 in SkBr3 cells (**E**) and CAFs (**F**) transfected for 48 h with 25 nM miR-Ctrl or miR-338-3p mimic (miR-338-3p m) in combination or not with 50 nM miR-338-3p inhibitor (miR-338-3p i) before treatment for 8 h with 100 nM E2 or 100 nM G-1. Each column represents the mean ± SD of three independent experiments performed in triplicate. (*) indicates *p* < 0.05 for cells receiving treatments vs cells treated with vehicle (-).

**Figure 8:**
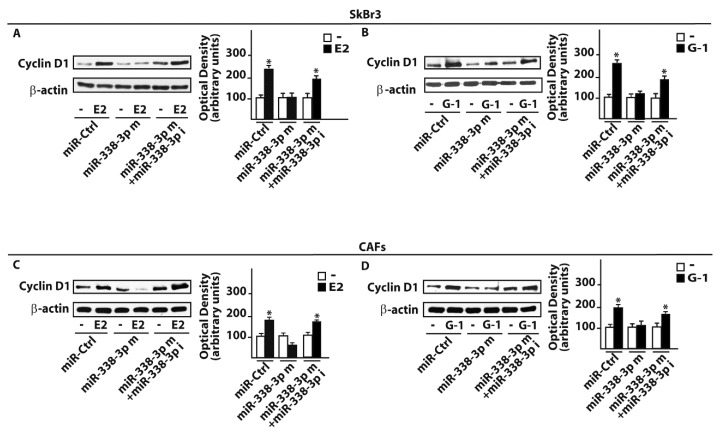
miR-338-3p prevents Cyclin D1 protein induction by E2 and G1 in SkBr3 cancer cells and CAFs. Cyclin D1 protein expression in SkBr3 cancer cells (**A**,**B**) and CAFs (**C**,**D**) transfected for 48 h with 25 nM miR-Ctrl or miR-338-3p mimic (miR-338-3p m) in combination or not with 50 nM miR-338-3p inhibitor (miR-338-3p i) before treatment for 12h with 100 nM E2 or 100 nM G-1. Side panels show densitometry analysis of the blots normalized to the loading control β-actin. (*) indicates *p* < 0.05 for cells receiving treatments vs cells treated with vehicle (-).

**Figure 9:**
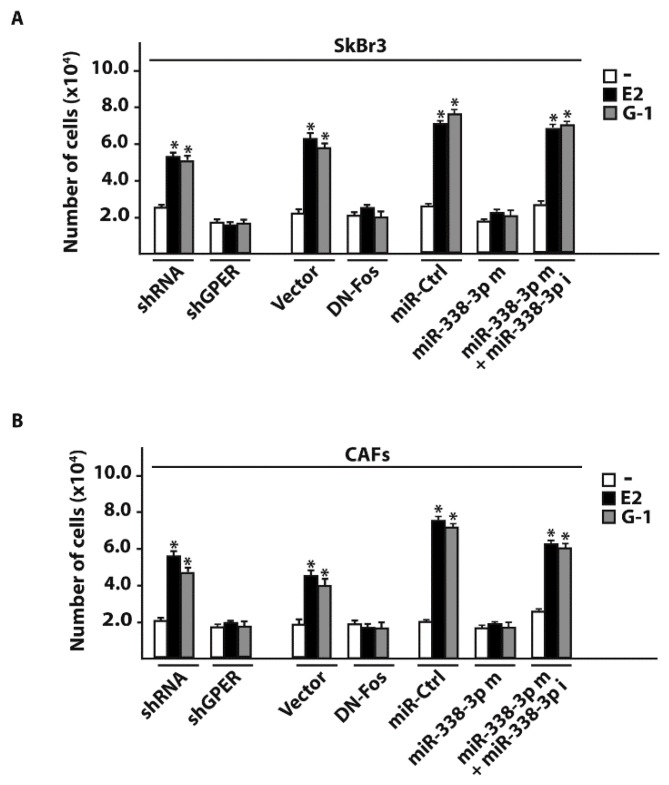

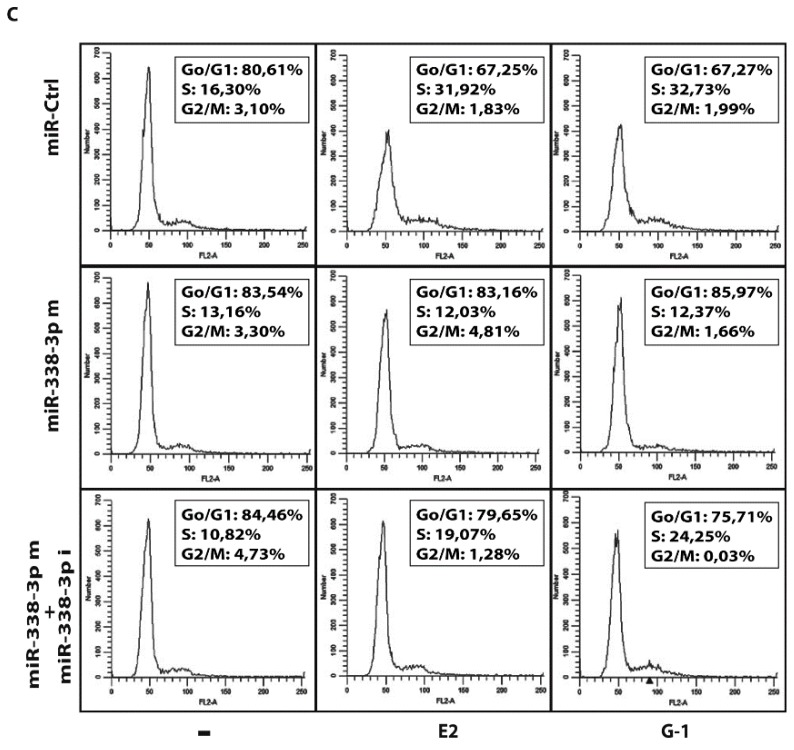
miR-338-3p decreases the proliferation of SkBr3 cancer cells and CAFs induced by E2 and G-1. Cell proliferation in SkBr3 cancer cells (**A**) and CAFs (**B**) transfected every 2 days with 100ng shRNA or shGPER, 100ng vector or a dominant-negative c-Fos construct (DN-Fos) and 25 nM miR-Ctrl or miR-338-3p mimic (miR-338-3p m) in combination or not with 50 nM miR-338-3p inhibitor (miR-338-3p i). Cells were treated every day with 100 nM E2 or 100 nM G-1 and counted on day 6. Each column represents the mean ± SD of three independent experiments performed in triplicate. (*) indicates *p* < 0.05 for cells receiving treatments vs cells treated with vehicle (-). (**C**) Representative pictures of cell cycle analysis in CAFs transfected for 48 h with 25 nM miR-Ctrl or miR-338-3p mimic (miR-338-3p m) in combination or not with 50 nM miR-338-3p inhibitor (miR-338-3p i) before the treatment for 24 h with 100 nM E2 and 100 nM G-1. In each panel, the percentages of cells in G0/G1, S and G2/M phases of the cell cycle are indicated. Values represent the mean ± SD of three independent experiments.

As in our previous investigations c-Fos was involved in the regulation of cyclins [43,45], we assessed that the transactivation of the Cyclin D1 promoter sequence by 100 nM E2 and 100 nM G-1 was prevented co-transfecting a dominant negative c-Fos expression construct (DN-Fos) in SkBr3 and CAFs (Figure 7A,B). Nicely recapitulating the aforementioned results, the Cyclin D1 promoter luciferase activity induced by 100 nM E2 and 100 nM G-1 was inhibited using the miR-338-3p mimic, an effect rescued by the miR-338-3p inhibitor sequence (Figure 7C,D). In addition, similar findings were observed evaluating the regulation of Cyclin D1 at both mRNA (Figure 7E,F) and protein levels (Figure 8A–D). As biological counterpart, the proliferative responses elicited by 100 nM E2 and 100 nM G-1 in SkBr3 cancer cells and CAFs were prevented silencing GPER or transfecting the DN-Fos construct (Figure 9A,B). Furthermore, the miR-338-3p mimic sequence decreased the proliferation induced by 100 nM E2 and 100 nM G-1 (Figure 9A,B), however this effect was rescued co-transfecting the miR-338-3p inhibitor (Figure 9A,B). Further supporting the aforementioned findings, the treatment for 24 h with 100 nM E2 and 100 nM G-1 triggered inhibitory effects on cell cycle progression transfecting CAFs with the miR-338-3p mimic sequence, however this response was rescued in the presence of the miR-338-3p inhibitor sequence (Figure 9C). Overall, these results suggest that estrogenic GPER signaling regulates miR-338-3p expression and function in SkBr3 cancer cells and CAFs.

## 4. Discussion

Performing a microarray analysis of 754 miRNAs involved in diverse diseases, in the present study we determined that diverse miRNAs are regulated by E2 in both SkBr3 breast cancer cells and CAFs. In particular, we assessed that E2 increases 23 miRNAs and lowers 2 miRNAs in SkBr3 cells, while E2 triggers the up-regulation of 7 miRNAs and the down-regulation of 22 miRNAs in CAFs. In addition, in both cell types E2 induced the expression of miR-144 and repressed the levels of miR-338-3p, which is known as an inhibitor of cancer progression [30,31,32,33,34]. Considering that miR-144 was investigated in our previous study [25], we attempted to provide novel insights into the estrogen regulation of miR-338-3p. First, we performed a METABRIC analysis that revealed a positive correlation of miR-338-3p with the overall survival in breast cancer patients. Then, we evidenced that a miR-338-3p mimic sequence prevents the expression of c-Fos, Cyclin-D1 and the growth effects induced by E2 and G-1 through GPER in SkBr3 cells and CAFs. Worthy, these effects triggered by E2 and G-1 were rescued using a miR-338-3p inhibitor sequence. Altogether, the aforementioned results provide new insights on the molecular mechanisms involved in the expression and function of certain miRNAs upon estrogen exposure in both breast cancer cells and CAFs.

Breast tumor is the most common malignancy in females and its incidence is increasing worldwide [46]. Several studies are ongoing in order to identify novels biological targets that may be considered toward innovative therapeutic approaches. To date, few markers like the estrogen receptor (ER), the progesterone receptor (PR) and the human epidermal growth factor receptor 2 (HER2), have been identified as predictors of clinical responses to breast cancer treatments [47]. None of these markers, however, well evaluates tumor invasion or provides early detection of cancer progression [48]. In this context, GPER has been suggested as a further predictor of breast cancer aggressiveness as its expression was found positively associated with clinic-pathological features of cancer progression and poor survival rates [49,50]. Moreover, GPER has been also indicated as an independent factor to predict a reduced disease-free survival in patients treated with tamoxifen [49]. The lack of GPER in the plasma membrane was also related to excellent long-term prognosis in ER-positive breast tumors treated with tamoxifen, an observation that highlighted the potential importance of GPER expression in different cancer cell types [51].

Despite the stimulatory effects elicited by GPER on the growth of diverse cancer cells [3,4,5,6], high doses of the GPER agonist G-1 (≥1 μM) have been shown to exert an inhibitory action on the proliferation of certain cancer cell lines [52,53,54,55,56]. Therefore, the different biological responses mediated by GPER in distinct tumor cell contexts may depend on the receptor expression repertoire, the signaling pathways activated and other factors that remain to be fully elucidated.

The involvement of diverse miRNAs in breast cancer progression has been well established [6]. For instance, it has been reported that let-7d, miR-210 and miR-221 are down-regulated in the breast ductal carcinoma in situ and up-regulated following the invasive transition. Moreover, miR-9, miR-10b, miR-21, miR-29a, miR-155 and miR-373-520 family were found to promote the metastatic tumor dissemination [57]. Next, member of the let-7, miR-200, miR-34 and miR-125b families, were able to regulate the epithelial-mesenchymal transition in breast cancer [57]. According to the results obtained in the present investigation, previous studies have indicated that in diverse pathophysiological conditions, including breast cancer, the regulation of certain miRNAs by E2 may involve GPER activation [21,25,58,59]. It has been shown that GPER activation by estrogens stimulates a network of transduction pathways, which triggers key factors involved in cell growth, differentiation and transformation, like c-Fos [5,44,60,61]. The proto-oncogene c-Fos represents a prototypical “immediate early” gene since its expression is rapidly induced by different extracellular stimuli through the activation of the serine-threonine kinases of mitogen-activated protein kinase (MAPK) family [62,63]. The nuclear protein encoded by c-Fos interacts with Jun family members to form the heterodimeric activating protein-1 transcription factor complex (AP-1), which binds to TGAC/GTC/AA sequences (AP-1 responsive elements) located within the promoter sequences of target genes [62,64]. Many studies focusing on the oncogenic functions of c-Fos have demonstrated its involvement in tumor growth through the modulation of Cyclin D1, which is a nuclear regulatory subunit of the cyclin-dependent kinases (CDK)-4 and CDK-6 [65,66,67]. Nicely fitting with these data, we determined that in SkBr3 cancer cells and CAFs E2 and G-1 induce c-Fos and Cyclin D1 expression toward cell proliferation. According to the inhibitory function of miR-338-3p in certain cancer types [30,31,32,33,34], we also found that miR-338-3p abrogates the abovementioned effects triggered by E2 and G-1 in SkBr3 cells and in important components of the tumor microenvironment as CAFs [35,36]. In this regard, our data highlight additional mechanisms by which tumor cells and CAFs cooperate toward worse cancer features. Well-fitting with the present findings, it has been established that cancer development involves the functional interaction of malignant cells with the tumor microenvironment [68,69]. For instance, stromal cells like CAFs generate a dynamic signaling network through the secretion of growth factors and cytokines that stimulate the proliferation and dissemination of cancer cells [70,71]. In this context, the regulation of miR-338-3p shared by breast cancer cells and CAFs may be a further mechanism linking the estrogen stimulation of both the tumor microenvironment and tumor cells.

## 5. Conclusions

miRNAs target numerous genes involved in the cell growth and survival of diverse types of tumors, including breast cancer [72]. Therefore, changes in miRNAs expression may have a prognostic role along with a therapeutic perspective in cancer patients. Here, we have provided novel insights on the molecular mechanisms through which estrogenic GPER signaling in both breast cancer cells and CAFs lowers the expression of miR-338-3p, which has been reported to act as an inhibitor of cancer cell growth and invasion [30,31,32,33,34]. Further studies are needed to better define the functions of miR-338-3p and its usefulness in innovative therapeutic approaches in breast cancer patients.
